# The relationship between clinical nurses’ job stress and presenteeism: the mediating effect of psychological detachment

**DOI:** 10.3389/fpubh.2026.1806113

**Published:** 2026-04-15

**Authors:** Hua Mao, Die Chen, Na Li, Xiang Zeng

**Affiliations:** 1 Nursing Department Education and Training Section, Jianyang Traditional Chinese Medicine Hospital, Chengdu, China; 2 Department of Psychiatry, Jianyang Traditional Chinese Medicine Hospital, Chengdu, China; 3 Nursing Department, Chongqing JiangJin District Hospital of Chinese Medicine, Chongqing, China

**Keywords:** China, job stress, nurse, presenteeism, psychological detachment

## Abstract

**Objective:**

This study aims to explore whether psychological detachment mediates the relationship between job stress and presenteeism among clinical nurses, and to determine the extent of the interaction between job stress, psychological detachment, and presenteeism.

**Methods:**

This study employed a convenience sampling method to select clinical nurses from five hospitals in Sichuan Province as the subjects of the investigation. A questionnaire survey was conducted using a general information survey form, the Chinese Nurses Stressor Scale (CNSS), the Psychological Detachment Questionnaire (PDQ), and the Stanford Presenteeism Scale-6 (SPS-6).

**Results:**

A total of 325 valid questionnaires were collected. The mean scores for the CNSS, PDQ, and SPS-6 were (94.64 ± 15.89), (9.76 ± 3.60), and (21.01 ± 5.42), respectively. A positive correlation was observed between clinical nurses’ presenteeism and job stress, while a significant negative correlation was found with psychological detachment (all *p* < 0.05). Job stress positively predicted presenteeism, whereas psychological detachment negatively influences presenteeism. Furthermore, psychological detachment partially mediates the relationship between job stress and presenteeism among clinical nurses, with an indirect effect of 0.06, accounting for 30% of the total effect.

**Conclusion:**

The findings indicate that job stress exerts both direct and indirect effects on the presenteeism of clinical nurses, with psychological detachment serving as a partial mediating factor that attenuates nurses’ levels of presenteeism.

## 1 Introduction

In recent years, reports of ‘death from overwork’ have become increasingly prevalent, capturing the attention of researchers regarding health behaviors and igniting debates surrounding the phenomenon of working while ill. This issue has emerged as a prominent topic in contemporary society (1). Presenteeism is defined as the behavior of employees who persist in working despite experiencing poor physical or mental health, leading to diminished work efficiency and productivity losses (2). It is important to note that not all attendance under compromised conditions constitutes health-related presenteeism. A proportion of such attendance decisions are primarily driven by structural and organizational pressures—including workforce shortages, inflexible shift arrangements, and institutional cultures that discourage absenteeism—rather than by employees’ health status per se (3). The present study focuses specifically on health-related presenteeism, defined as the productivity loss incurred when employees attend work despite experiencing genuine physical or mental health impairment.

Evidence indicates that presenteeism is particularly severe in the nursing industry (4, 5). For instance, prevalence of presenteeism of nurses at work is 36.5% in Switzerland (6), 78.4% in the United States (7), and as high as 94.3% in China (8). Chronic work overload, insufficient organizational resources, work-family imbalance, high rates of occupational burnout, and low career satisfaction contribute to nurses’ susceptibility to work participation issues (9, 10). This phenomenon not only increases the likelihood of adverse patient outcomes, such as medication errors and patient falls (11, 12), but has also been confirmed to impose an economic burden on healthcare organizations globally (13). The quality of clinical nursing is closely linked to patient treatment outcomes, rehabilitation processes, and healthcare experiences, making the work participation of clinical nurses crucial to the quality of clinical nursing (14). Understanding the work participation of clinical nurses is a key step in enhancing nursing service quality, maximizing nursing productivity, and expanding the scope of practice. However, research on presenteeism among clinical nurses is limited, both in China and internationally. Thus, investigating the factors influencing presenteeism among clinical nurses in China is essential for maintaining a healthy and motivated clinical nursing workforce.

The key factor related to nurses’ presenteeism is job stress. Currently, the global population is rapidly expanding, which presents challenges for the nursing industry, particularly regarding workforce shortages (15). By the end of 2021, the number of nurses in China reached 5.018 million, yielding a registered nurse-to-population ratio of 3.56 per thousand, which falls short of the global average of 3.69 per thousand recorded in 2018 (16). The high workload and time constraints resulting from insufficient human resources reflect the significant quantifiable work demands placed on clinical nurses (17). Furthermore, frequent emergency care operations and shift work exacerbate the psychological workload of clinical nurses, leading to increased job stress (18). Previous studies have indicated that excessive work conditions significantly contribute to the incidence of presenteeism among employees (19, 20). When employees experience excessive job stress, the resultant imbalance between work and family, coupled with low occupational well-being, can lead to role overload and internal role conflict, negatively impacting employees’ physical and mental health (e.g., occupational burnout, emotional exhaustion, and presenteeism). Therefore, we propose Hypothesis 1: job stress has a positive impact on the presenteeism rate of clinical nurses.

Persistent work-related stress can lead to intrusive thoughts about work, characterized by continuous rumination on work-related mistakes or the anticipation of potential risks during non-working hours. This psychological burden transcends temporal and spatial boundaries, severely hindering the capacity for psychological detachment—specifically, the ability to sever cognitive and emotional ties to professional activities in non-work settings (21). Research (22) indicates that when nurses achieve a higher level of psychological detachment, the relationship between work-related stress and physical fatigue, mental fatigue, and sleep quality is significantly weakened. It should be noted, however, that job stress does not uniformly lead to negative outcomes. Li et al. (23) demonstrated, drawing on activation theory and Conservation of Resources Theory, an inverted U-shaped relationship between job stress and performance, suggesting that moderate stress may stimulate adaptive behaviors, whereas excessive or uncontrollable stress depletes psychological resources and impairs individual functioning. In the clinical nursing context, the stressors captured by the Chinese Nurses Stressor Scale, including uncontrollable workload surges, emergency care demands, role conflicts, and insufficient organizational support, are predominantly hindrance-type stressors in nature. Unlike challenge stressors that may be motivating and contained within the workday, hindrance stressors offer no compensatory developmental returns; instead, they impose sustained cognitive and emotional burdens that frequently extend beyond working hours in the form of intrusive work-related rumination, thereby eroding nurses’ capacity for off-duty recovery (24). Therefore, we propose Hypothesis 2: Work-related stress negatively impacts the psychological detachment of clinical nurses.

As research on the impact of job stress on mental health and work behavior gains prominence, recovery experiences are increasingly recognized as a key mechanism underlying these interactions. Previous studies have indicated a negative correlation between recovery experiences and presenteeism rates (17, 25). Psychological detachment, a core component of work recovery, refers to an individual’s physical and psychological separation from work during non-working hours (24, 26), significantly influencing employees’ presenteeism rates. Specifically, Huyghebaert-Zouaghi et al. (27) found that employees’ psychological detachment is negatively correlated with presenteeism rates. When employees frequently encounter high work demands, their physical and mental functions remain activated. If they cannot fully detach from work to restore their physical and mental stress to baseline levels, prolonged low relaxation experiences and high emotional exhaustion may lead to negative outcomes, such as sleep disorders, low work engagement, and attendance issues (28). Furthermore, Cho et al. (22) discovered that high levels of psychological detachment weaken the association between job stress and physical fatigue, mental fatigue, and sleep quality, with psychological detachment mediating the relationship between workload and fatigue and sleep problems. Related research (29) indicates that an adequate level of psychological detachment enables nurses to effectively separate from work during non-working hours, helping to alleviate Job stress, reduce burnout, and improve their quality of life, thus allowing them to recover their energy and better cope with work challenges. Therefore, we propose Hypothesis 3: Psychological detachment negatively impacts presenteeism rates among clinical nurses.

**Figure 1:**
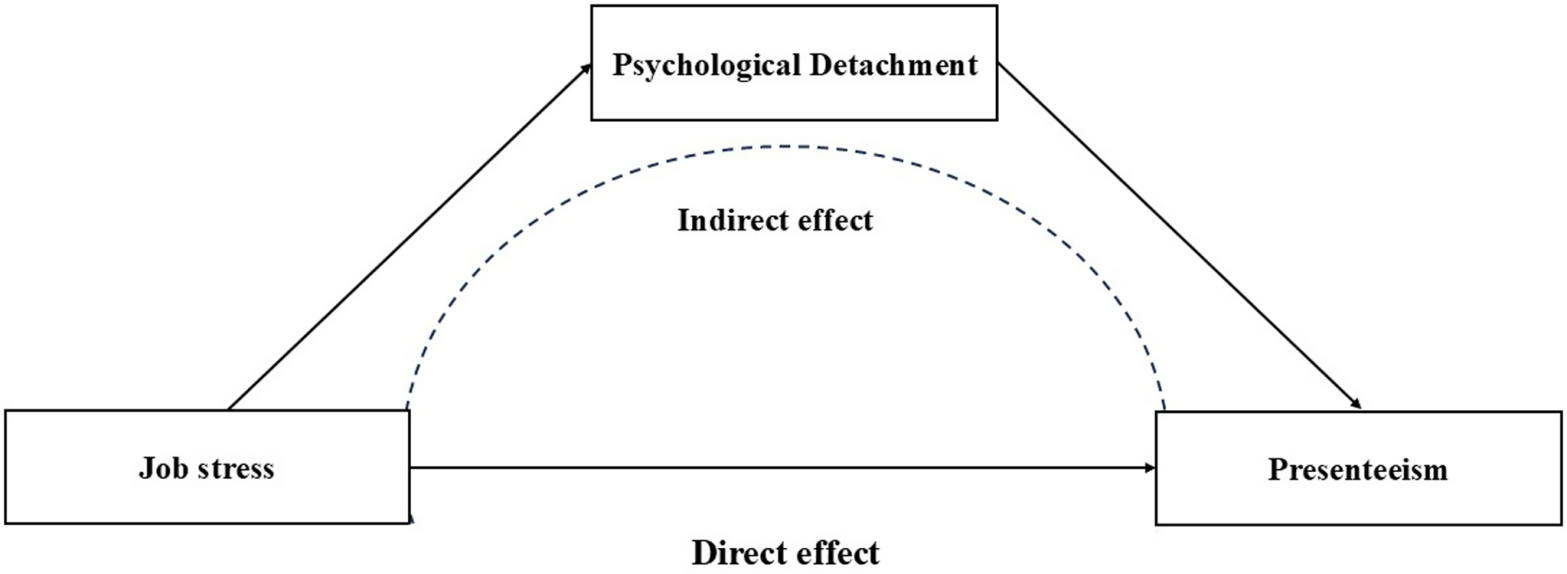
The relationship between job stress, psychological detachment, and presenteeism in this study.

Conservation of Resources Theory posits that individuals are inherently motivated to acquire, retain, and protect valued resources, and that the actual or threatened loss of such resources constitutes a primary source of psychological stress (30). In the clinical nursing context, hindrance stressors—including work overload, role conflict, and insufficient organizational support—are associated with sustained depletion of nurses’ cognitive and emotional resources, thereby reducing their available psychological resource reserves. This progressive net resource loss not only undermines self-regulatory capacity but also impairs the individual’s ability to transition from a state of work-related activation to a restorative state during non-working hours, manifesting as reduced levels of psychological detachment. Building on this resource depletion logic, Effort-Recovery Theory further elucidates the mechanism through which psychological detachment shapes health outcomes (31). The theory specifies that sustained occupational effort generates load reactions—including fatigue accumulation, negative affect, and cognitive impairment—that can only be effectively resolved when the individual achieves genuine psychological detachment following work cessation, thereby halting work-related cognitive and affective activation and enabling the gradual recovery of physiological and psychological resources. When psychological detachment is compromised, nurses may remain in a state of work-related cognitive activation and affective arousal during off-duty hours, impeding the recovery process and allowing load reactions to accumulate across successive work cycles—a pattern that is significantly associated with the occurrence of presenteeism behaviors. According to E-R Theory, sustained psychological activation—arising from prolonged exposure to high-intensity work demands such as overtime and shift work—combined with cognitive stress processes such as rumination, constitutes a dual impediment to the recovery process (32). Relevant evidence indicates that prolonged direct or indirect exposure to occupational stressors can lead to increased psychological distress—including anxiety, emotional suffering, and depression—as well as physical symptoms such as insomnia and fatigue among clinical nurses, ultimately impairing their work efficiency. Several qualitative (33–35) studies similarly highlight that when confronted with complex and challenging clinical care situations, clinical nurses may experience self-doubt, ruminative thinking, and professional alienation; the repeated recollection of stressful scenarios prevents full disengagement from these stressors. In sum, COR Theory explicates the resource depletion pathway linking job stress to impaired psychological detachment, while E-R Theory illuminates the recovery-disruption mechanism through which diminished psychological detachment contributes to presenteeism. The two theories are conceptually complementary and jointly provide the theoretical rationale for the proposed sequential pathway from job stress through psychological detachment to presenteeism. Accordingly, we propose the final hypothesis: Hypothesis 4: Psychological detachment mediates the relationship between job stress and presenteeism among clinical nurses. The hypothesized model is depicted in Figure 1.

## 2 Methods

### 2.1 Research subjects

This study selected clinical nurses from the hospitals in Jianyang City, Sichuan Province, using convenience sampling from November 2025 to January 2026. The inclusion criteria for this study were as follows: (1) engaged in clinical nursing work for at least 6 months; (2) possessing a nursing practice certificate. The exclusion criteria were: (1) nurses who do not directly contact patients, such as those in sterilization supply rooms and infusion centers; (2) those who are absent from work for more than 3 months during the study period; (3) those who have experienced significant life events in the past 3 months (e.g., divorce, death of a spouse, child, or parent, or serious illness). The sample size for this cross-sectional study should be at least 5 to 10 times the number of study variables and the dimensions of the scales. This study involves 12 demographic variables, with the Chinese Nurses Stressor Scale comprising 5 dimensions, the psychological detachment questionnaire consisting of 1 dimension, and the Stanford Presenteeism Scale-6 containing 2 dimensions, totaling 8 dimensions. The sample size should be between 100 and 200, and considering a 20% invalid questionnaire recovery rate, the total sample size required for this study is 125 to 250 cases.

### 2.2 Research tools

#### 2.2.1 General demographic information

A self-designed questionnaire developed by the research team was utilized to gather data on various demographic factors, including hospital level, gender, age, marital status, personal monthly income, frequency of weekly physical exercise, professional title, self-rated health status, interpersonal relationships, department, years in position, and daily sleep duration.

#### 2.2.2 Chinese nurses stressor scale (CNSS)

The measurement of Job stressors among nurses is conducted using the Chinese Nurses Stressor Scale (CNSS), which was revised by Li and Liu (36). This scale encompasses five dimensions with a total of 35 items: ① Issues related to the nursing profession and work, consisting of 7 items aimed at assessing the professional challenges faced in nursing practice; ② Issues concerning workload and time allocation, comprising 5 items intended to evaluate workload pressure and the rationality of time distribution in relation to task volume; ③ Issues regarding the work environment and resources, including 3 items designed to assess environmental equipment pressure and examine the impact of working conditions on practice; ④ Issues related to patient care, with 11 items aimed at evaluating patient care pressure, reflecting the difficulties encountered during the clinical nursing implementation process; ⑤ Issues concerning management and interpersonal relationships, consisting of 9 items intended to assess management interpersonal pressure, focusing on organizational management and interpersonal interaction factors. The scale employs a 4-point Likert scoring method, with scores ranging from 1 to 4, corresponding to ‘strongly disagree’ to ‘strongly agree’. The total score ranges from 35 to 140, with higher scores indicating greater Job stress among nurses. Specifically, a score of 35 to 70 indicates mild stress, 71 to 105 indicates moderate stress, and 106 to 140 indicates severe stress, with higher total scores suggesting a higher level of Job stress among nurses. This scale has a broad foundational application in nursing research within China, with validity tests indicating that the Cronbach’s *α* coefficient reached 0.981. In this study, the Cronbach’s α coefficient for this scale was found to be 0.912.

#### 2.2.3 Psychological detachment questionnaire (PDQ)

The level of psychological detachment among nurses was assessed using the Psychological Detachment Questionnaire from the Recovery Experience Questionnaire developed by Sonnentag and Fritz (31). This scale features a unidimensional structure and comprises four items, employing a 5-point Likert scale (1 to 5). The total score ranges from 4 to 20, with higher scores indicating a greater degree of psychological detachment. According to conventional research standards, a median total score of 11 points was established as the cutoff for categorizing participants into high and low psychological detachment groups. The original scale exhibited a Cronbach’s *α* coefficient of 0.84 and a KMO value of 0.74, demonstrating good reliability and validity, as well as robust psychometric properties, and has been extensively utilized in related research fields. Scholars such as Lu et al. (37) have adapted the scale for Chinese contexts and validated its applicability among nursing populations, reporting a Cronbach’s *α* coefficient of 0.833. In this study, the Cronbach’s α coefficient for this scale was found to be 0.811.

#### 2.2.4 Stanford Presenteeism Scale-6 (SPS-6)

The Stanford Presenteeism Scale-6 (SPS-6) was developed by Koopman et al. at Stanford University in the United States (38) and subsequently translated and revised by Zhao et al. in 2010 (39). The version utilized in this study is the one revised and translated by Zhao Fang et al., which demonstrates a Cronbach’s *α* coefficient of 0.760 and a KMO statistic of 0.745. This scale comprises six items that encompass two dimensions: work limitations (four items) and work engagement (two items). A 5-point Likert scale is employed, where 1 indicates ‘strongly disagree’ and 5 indicates ‘strongly agree,’ with items 5 and 6 requiring reverse scoring. The total score ranges from 6 to 30, with higher scores indicating a more severe level of presenteeism, reflecting greater productivity loss due to health issues. In this study, the Cronbach’s *α* coefficient for this scale was found to be 0.875.

### 2.3 Ethical review

This study adheres to the ethical guidelines established in the Declaration of Helsinki (1964). Written permission for this research has been granted by the Ethics Committee of the Traditional Chinese Medicine Hospital of Jianyang, Sichuan Province (Approval No. Jianzhongyi Lun-2026-01).

### 2.4 Data collection

All items were entered into the Wenjuanxing platform by the researcher, who distributed the electronic questionnaire to individuals via the WeChat platform. The purpose and significance of the study were clearly articulated on the homepage of the questionnaire, emphasizing that the research was conducted anonymously. Participants could only commence filling out the questionnaire after selecting ‘Agree to Participate.’ All items were designated as mandatory to prevent missing responses. Following the completion of the questionnaire collection, the researcher screened the responses for any significant patterns or illogical anomalies to ensure data quality.

### 2.5 Data analysis

The data obtained were analyzed using IBM SPSS version 26.0 and the PROCESS macro version 5.0. Frequency and percentage analyses were employed to describe the general demographic characteristics of the study, while continuous variables were represented by means and standard deviations. Independent samples t-tests and one-way analysis of variance (ANOVA) were utilized to determine differences in presenteeism among clinical nurses based on various demographic factors. Pearson correlation coefficients were calculated to examine the relationships between presenteeism and two key variables: job stress and psychological detachment among clinical nurses. To further elucidate these interrelationships, multiple linear regression analyses were conducted with presenteeism scores as the dependent variable, categorized into three distinct levels. The first model included demographic variables (hospital grade, gender, age, personal monthly income, weekly exercise frequency, interpersonal relationships, department, and daily sleep duration) as control parameters. The second model added job stress scores as an additional independent variable based on the first model, while the third model introduced the variable of psychological detachment. To assess the potential threat of common method bias (CMB) arising from the use of self-reported questionnaire data collected at a single time point, Harman’s single-factor test was conducted. All items measuring the study variables were simultaneously entered into an exploratory factor analysis without rotation. Although Harman’s single-factor test has recognized limitations in its sensitivity to detect common method bias, procedural remedies were also implemented during data collection, including guaranteed respondent anonymity and explicit instructions that there were no right or wrong answers, so as to further mitigate the potential influence of common method bias.

## 3 Results

In this study, a total of 340 questionnaires were distributed, and 330 were returned. After excluding 10 invalid questionnaires—5 discarded due to a response time of less than 100 s or due to discernible patterns in the answers—a final count of 325 valid questionnaires was included, resulting in a valid response rate of 95.59%. Prior to the main analyses, Harman’s single-factor test was performed to evaluate the potential threat of common method bias. The results indicated that 12 factors with eigenvalues greater than 1.0 were extracted, with the first factor accounting for 25.72% of the total variance—well below the recommended threshold of 40%. As no single factor explained the majority of the variance, common method bias was deemed not to pose a significant threat to the validity of the present findings.

### 3.1 Descriptive statistics of variables

The composite mean scores and corresponding standard deviations (SD) for job stress, psychological detachment, and presenteeism are (94.64 ± 15.89), (9.76 ± 3.60), and (21.01 ± 5.42), respectively. Refer to Table 1 for further details.

**Table 1:** Scores of each dimension of the scales (*n* = 325).

Variables	Range of scores	$\bar{\chi }$ ± SD
CNSS	50 ~ 120	94.64 ± 15.89
1	7 ~ 28	19.02 ± 4.30
2	5 ~ 20	13.52 ± 3.17
3	3 ~ 12	8.11 ± 2.07
4	11 ~ 40	29.99 ± 6.92
5	9 ~ 36	24.01 ± 5.49
PDQ	4 ~ 20	9.76 ± 3.60
SPS-6	6 ~ 30	21.01 ± 5.42
1	4 ~ 20	13.98 ± 3.66
2	2 ~ 10	7.03 ± 1.97

### 3.2 Participant characteristics

Table 2 presents the results of mean differences for the selected demographic variables along with their statistical significance.

**Table 2:** Participants’ sociodemographic data and univariate analysis of SPS-6 (*N* = 325).

Variables	*N* (%)	SPS-6 (SD)	*t/F*	*p*
Hospital level			4.006	<0.001
1	48 (14.77)	18.79 ± 6.76		
2	71 (21.85)	20.14 ± 4.69		
3	206 (63.38)	21.82 ± 5.13		
Gender			76.55	<0.001
Male	45 (13.85)	15.09 ± 6.10		
Female	280 (86.15)	21.96 ± 4.66		
Age			6.948	<0.001
20–30 years	126 (38.77)	19.40 ± 5.89		
31–40 years	105 (32.31)	21.70 ± 4.86		
41–50 years	56 (17.23)	22.84 ± 4.69		
>50 years	38 (11.69)	21.71 ± 5.02		
Marital status			1.491	0.227
Unmarried	93 (28.62)	21.80 ± 4.90		
Married	200 (61.54)	20.63 ± 5.70		
Divorce	32 (9.84)	21.09 ± 4.94		
Monthly income			11.65	<0.001
<3,000 RMB per month	36 (11.08)	22.11 ± 5.31		
3,000–5,000 RMB per month	115 (35.38)	22.82 ± 3.43		
5,001–8,000 RMB per month	122 (37.54)	20.20 ± 5.34		
>8,000 RMB per month	52 (16.00)	18.12 ± 7.41		
Frequency of weekly physical activity sessions			6.07	0.003
1 ~ 3	147 (45.23)	22.12 ± 4.63		
4 ~ 6	105 (32.31)	20.33 ± 5.64		
>6	73 (22.46)	19.74 ± 6.15		
Professional title			0.734	0.533
Nurse	124 (38.15)	21.32 ± 5.47		
The primary nurse practitioner	85 (26.15)	20.36 ± 5.51		
Nurse-in-charge	65 (20.00)	20.82 ± 5.17		
Deputy director nurse or above	51 (15.69)	21.01 ± 5.42		
Self-assessment of Health Status			0.064	0.938
Bad	58 (17.85)	20.98 ± 5.21		
Normal	150 (46.15)	20.91 ± 5.29		
Good	117 (36.00)	21.15 ± 5.71		
Employment status			7.898	0.005
Permanent staff	74 (22.77)	22.55 ± 4.72		
Temporary contract staff	251 (77.23)	20.55 ± 5.53		
Department			6.284	<0.001
Internal medicine	83 (25.54)	19.52 ± 5.69		
Surgery	92 (28.31)	19.86 ± 5.91		
Obstetrics and gynecology	53 (16.31)	21.21 ± 5.10		
Pediatrics	42 (12.92)	22.40 ± 3.63		
Emergency medicine	27 (8.31)	23.85 ± 4.24		
ICU	28 (8.62)	23.96 ± 4.13		
Years in position			1.295	0.275
1 ~ 5 years	133 (40.92)	20.43 ± 5.36		
6 ~ 10 years	108 (33.23)	21.46 ± 5.29		
≥10 years	84 (25.85)	21.33 ± 5.42		
Daily sleep duration			8.251	<0.001
≤6 h	103 (31.69)	22.57 ± 5.04		
6 ~ 8 h	123 (37.85)	20.86 ± 5.11		
≥8 h	99 (30.46)	19.56 ± 5.77		

### 3.3 Factors related to presenteeism

The results of the univariate analysis indicate significant differences across various factors, including hospital level, gender, age, monthly income, frequency of weekly physical activity sessions, professional title, self-assessment of health status, employment status, department, years in position and daily sleep duration. To further investigate the relationships among these variables, we conducted a multiple hierarchical regression analysis. In this analysis, we included statistically significant factors from the univariate analysis as control variables. Job stress and psychological detachment were designated as predictor variables, while presenteeism served as the dependent variable. The statistical analysis demonstrated a good model fit, and the Variance Inflation Factor (VIF)—a regression diagnostic tool used to assess the severity of multicollinearity—was employed to evaluate potential multicollinearity. The results indicated that the VIF values ranged from 1.041 to 1.974, which is well below the standard threshold of 5. Furthermore, the tolerance values ranged from 0.507 to 0.960, all exceeding the minimum acceptable level of 0.1, indicating no multicollinearity issues among the independent variables. In Model 1, the demographic variables accounted for 37.2% of the variance in presenteeism. However, in this model, age and interpersonal relationships did not exhibit significance. Upon entering Model 2, we introduced job stress as a variable while controlling for the demographic characteristics established in Model 1. This addition significantly enhanced the explanatory power of the model, with job stress contributing an additional 10.8% to the variance in presenteeism.

In the final model (Model 3), we further refined our analysis by incorporating the concept of psychological detachment while maintaining control over the demographic characteristics and Job stress factors considered in the previous models. The inclusion of psychological detachment enhanced the model’s explanatory power by an additional 3.7%. Collectively, the variables across all three models accounted for 51.7% of the variance in the presenteeism of clinical nurses, as illustrated in Table 3.

**Table 3:** Multiple hierarchical regression analysis of sps-6 (*N* = 325).

Variables	Model 1				Model 2				Model 3			
	*β*	*P*	Tolerance	VIF	*β*	*P*	Tolerance	VIF	*β*	*P*	Tolerance	VIF
Hospital level	1.183	<0.001	0.96	1.041	0.51	0.102	0.893	1.12	0.436	0.147	0.891	1.123
Gender	5.118	<0.001	0.865	1.156	4.548	<0.001	0.856	1.169	3.943	<0.001	0.828	1.208
Age	0.168	0.554	0.684	1.463	−0.203	0.44	0.663	1.508	−0.145	0.568	0.662	1.511
Monthly income	−0.92	0.005	0.668	1.496	−0.702	0.02	0.663	1.508	−0.659	0.024	0.662	1.509
Frequency of weekly physical activity sessions	−1.435	<0.001	0.916	1.092	−0.512	0.097	0.793	1.261	−0.467	0.116	0.792	1.262
Employment status	−0.568	0.337	0.925	1.081	−0.277	0.607	0.921	1.086	−0.137	0.792	0.918	1.089
Department	0.729	<0.001	0.919	1.088	0.507	0.001	0.888	1.127	0.444	0.002	0.881	1.136
Daily sleep duration	−1.666	<0.001	0.89	1.123	−0.541	0.094	0.728	1.374	−0.493	0.114	0.727	1.375
Job stress					0.144	<0.001	0.606	1.650	0.106	<0.001	0.507	1.974
Psychological detachment									−0.359	<0.001	0.664	1.507
*F*(*P*)	24.956 (<0.001)				34.236 (<0.001)				35.719 (<0.001)			
*R^2^*	0.387				0.494				0.532			
*R^2^* change	0.372				0.480				0.517			

### 3.4 Correlation between variables

The effect sizes of the correlations among presenteeism, job stress and psychological detachment in the path model were moderate (*r* = −0.544, *r* = 0.594, *r* = −0.554). All variables exhibited significant associations (*p* < 0.01), as illustrated in Table 4.

**Table 4:** Pearson correlation among study variables (*N* = 325).

Variables	Job stress	Psychological detachment	Presenteeism
Job stress	1		
Psychological detachment	−0.544^a^	1	
Presenteeism	0.594^a^	−0.554^a^	1

^a^*p* < 0.001.

### 3.5 Examination of the mediating effect

**Figure 2:**
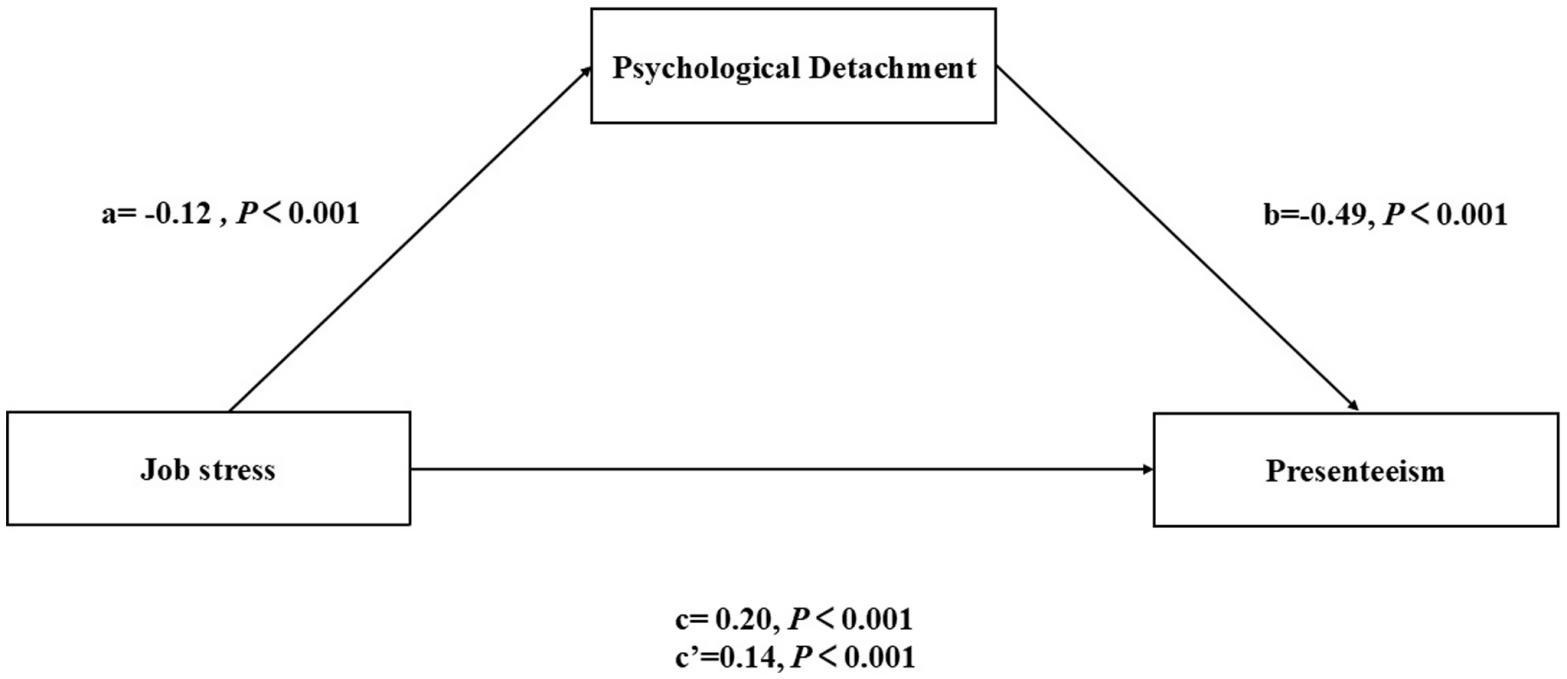
The mediating effect of psychological detachment between job stress and presenteeism.

The results indicate that Job stress has a significant positive predictive effect on presenteeism (*β* = 0.14, t = 8.28, *p* < 0.001). Conversely, job stress negatively predicts psychological detachment (*β* = −0.12, *p* < 0.001), while psychological detachment negatively predicts presenteeism (*β* = −0.49, *p* < 0.001). This suggests that psychological detachment serves as a partial mediator between job stress and presenteeism, with a mediating effect value of (*β* = 0.06, *p* < 0.001). The total effect is quantified at 0.20 (*p* < 0.001). To test the mediating effect, the Bootstrap procedure was employed, utilizing 5,000 resamples. The 95% confidence interval for the mediating effect of psychological resilience, 95%CI (0.04 ~ 0.09), does not include 0, thereby validating the mediating effect model, which accounts for 30% of the total effect. Detailed information can be found in Table 3, while Table 5 lists the specific effect values for each path. Figure 2 illustrates the specific pathways within this model.

**Table 5:** The efects of each path in the mediation mode (*N* = 325).

Effect	Path	*β*	SE	*t*	*p*	95%CI
Direct effect	X → Y	0.14	0.02	8.28	<0.001	(0.11, 0.18)
	X → M	−0.12	0.01	−11.65	<0.001	(−0.14, −0.10)
	M → Y	−0.49	0.08	−6.54	<0.001	(−0.64, −0.35)
Indirect effect	X → M → Y	0.06	0.01	-	-	(0.04, 0.09)
Total effect	X → M → Y	0.20	0.02	13.27	<0.001	(0.17, 0.23)

X, job stress; M, psychological detachment; Y, presenteeism; SE, standard error; CI, confidence interval.

## 4 Discussion

This study aims to explore the mediating effect of psychological detachment among clinical nurses regarding the relationship between job stress and presenteeism. The results indicate that psychological detachment not only directly affects presenteeism but also exerts an indirect influence on it. The total mediating effect is 30%, suggesting that psychological detachment serves as a significant mediating variable in elucidating the relationship between job stress and presenteeism.

### 4.1 Job stress, psychological detachment, and Presenteeism scores

In this study, the job stress score of nurses was found to be (94.64 ± 15.89), indicating a level above the moderate threshold. This finding aligns with the results reported by Wang et al. (40). The elevated stress levels may be attributed to the significant imbalance between the number of hospital beds and the nursing workforce in our country. The average patient load per nurse considerably exceeds the reasonable workload, leading to an overwhelming daily burden. In addition to fulfilling established responsibilities such as treatment, basic care, and nursing documentation in accordance with medical orders, nurses are also required to respond to sudden changes in patient conditions and emergencies, including resuscitation efforts. Moreover, the ongoing promotion of high-quality nursing services has heightened expectations and demands from patients, families, and healthcare institutions regarding nursing quality. This necessitates that nurses dedicate additional effort to technical operations, health education, personalized care, and continuity of care. Consequently, under the strain of multiple stressors, nurses experience substantial pressure concerning their workload and time management, resulting in prolonged periods of high stress and heavy load. This situation not only jeopardizes their physical and mental well-being but may also hinder the refinement and safety of nursing services.

In this study, the psychological detachment score of nurses was found to be (9.76 ± 3.60), which is considered low compared to the median score of 11. Nursing work typically involves long shifts, high-intensity physical labor, and frequent emotional exhaustion. Particularly when dealing with critically ill patients or emergency situations, nurses are required to maintain a high level of attention and professionalism, which may hinder their ability to completely detach psychologically from work after their shifts (38). Additionally, the support systems within the nursing work environment may significantly impact psychological detachment. For instance, insufficient colleague support, ineffective communication channels, and inadequate attention from management regarding nurses’ mental health can all diminish their ability to relax and recover psychologically after work. Qiao et al. (41) drawing on Terror Management Theory as a theoretical framework, demonstrated that cancer patients achieve psychological renewal through a dual-level defense mechanism during travel interventions: proximal defenses (e.g., sensory immersion, attentional distraction) facilitate temporary disengagement from disease-related stressors, while distal defenses (e.g., meaning reconstruction, self-esteem restoration) support deeper psychological integration — a process whereby individuals replenish depleted resources through transient cognitive-affective detachment from dominant stressors and subsequently return to functioning in a renewed state. Therefore, hospitals should prioritize the mental health of nursing staff by optimizing work processes, providing psychological support services, and improving the work environment. These measures could help nurses achieve better psychological detachment and enhance their job satisfaction and quality of life.

The results of this study indicate that the presenteeism score for clinical nurses is (21.01 ± 5.42), which is considered high. This finding aligns with the results reported by Min et al. (39) and exceeds the scores observed for general healthcare staff (40). The observed difference may be attributed to variations in study duration, participant demographic characteristics, and the evaluation methodologies employed. Furthermore, the prevalence of presenteeism among clinical nurses can negatively impact the quality of clinical care and increase vulnerability to psychological and physiological health issues. Therefore, addressing the issue of presenteeism among clinical nurses is of paramount importance, necessitating urgent intervention measures to enhance their attendance.

### 4.2 Correlation analysis between variables

This study demonstrates a positive correlation between job stress and presenteeism, consistent with the findings of Zhang et al. (42). This indicates that as Job stress increases, presenteeism also rises (Table 3), thereby confirming Hypothesis 1. The nature of nursing work is characterized by high intensity, high risk, and high pressure. Nurses often continue to work even when they are physically unwell or under stress due to heavy workloads and irreplaceable responsibilities, which leads to presenteeism (43). Furthermore, job stress can directly undermine nurses’ work capabilities and focus, consequently reducing their efficiency and productivity—core manifestations of presenteeism (44). Additionally, related research (45) has found that the atmosphere of presenteeism within teams interacts with job stress, further exacerbating presenteeism behaviors. Job stress plays a partial mediating role between the team atmosphere of presenteeism and presenteeism, indicating that stress is a significant transmission mechanism for presenteeism.

This study indicates a negative correlation between Job stress and psychological detachment, consistent with the findings of Wang et al. (46). It suggests that as job stress increases, nurses find it increasingly difficult to detach from their professional responsibilities during non-working hours, highlighting the significant impact of Job stress on individuals’ psychological recovery capacity. Research by Sagherian et al. (47) confirms that the recovery mechanism of nurses, which involves briefly detaching from work during rest periods, is often disrupted by excessive workloads, adversely affecting their physical and mental resource recovery. Furthermore, the study (31) emphasizes that high-intensity workloads and continuous emotional exhaustion can trigger cognitive rumination, making it challenging for individuals to disengage from work-related thoughts during their non-working hours.

This study demonstrates a negative correlation between psychological detachment and presenteeism, aligning with the findings of Jiang et al. (48). Nurses who possess stronger psychological detachment abilities exhibit lower levels of presenteeism, suggesting that effective psychological detachment can mitigate the occurrence of presenteeism; conversely, inadequate psychological detachment may exacerbate it. From the perspectives of the Conservation of Resources Theory and the Effort-Recovery Model, when nurses experience sustained resource depletion due to high work pressure and are unable to replenish their resources through psychological detachment, they may enter a ‘resource loss spiral’ (49). In this state, although nurses are physically present at work, their resource exhaustion may manifest as distraction, emotional fatigue, and physical depletion, resulting in presenteeism characterized by difficulties in engaging effectively in tasks. Psychological detachment enables nurses to safeguard their remaining resources by ceasing work-related thoughts and replenishing new resources through rest and social interactions. If the ability to psychologically detach is weak, resources cannot be replenished, compelling nurses to function in a ‘low battery mode,’ which significantly heightens the risk of presenteeism (47). Therefore, this suggests to nursing managers that facilitating effective detachment from work during non-working hours can aid nurses in restoring their physical and psychological resources through rest, leading to enhanced focus and efficiency while on duty, thereby reducing behaviors characterized as ‘being present but not productive.’ Nursing managers can provide mental health support, such as psychological counseling and stress management courses, and promote cultural initiatives, such as encouraging vacations, to facilitate nurses’ psychological detachment and subsequently reduce presenteeism.

### 4.3 The mediating role of psychological detachment between job stress and presenteeism

The results of this study elucidate the mediating role of psychological detachment between Job stress and presenteeism among clinical nurses, which supports Hypothesis 4. Specifically, job stress appears to influence presenteeism through two pathways: a direct pathway and an indirect pathway mediated by psychological detachment. On one hand, Job stress directly predicts the degree of presenteeism; on the other hand, it also indirectly affects presenteeism among clinical nurses through the mediating effect of psychological detachment. Sonnentag et al. (31) assert that individuals must exert effort to cope with work demands, and this effort depletes personal resources, leading to physiological responses such as fatigue. Short breaks can assist individuals in returning to a baseline state; during these breaks, work demands no longer impact individuals, and their responses to workload gradually diminish and eventually disappear. During periods of “psychological detachment” nurses can engage in activities unrelated to work, such as leisure, travel, exercise, and enjoying food, which allows them to acquire new resources and mitigate the depletion of psychological resources, thereby restoring their physical and mental states for a healthier return to work (50). Nursing managers implement psychological detachment training for nurses through various methods, including mindfulness courses and situational simulations (50). This training enables nurses to comprehend the significance of psychological detachment and equips them with techniques to foster it. Nursing managers may consider implementing psychological detachment training for nurses through methods such as mindfulness courses and situational simulations, which may help nurses better understand the value of psychological detachment and develop relevant coping techniques. Encouraging nurses to cultivate hobbies, engage in physical activity, and ensure adequate rest during non-working hours may further support their capacity to disengage from work-related demands. However, it should be noted that the indirect effect identified in this study (*β* = 0.06) is modest in magnitude, accounting for 30% of the total effect. This finding suggests that while psychological detachment represents a meaningful pathway linking job stress to presenteeism, it captures only a partial segment of the underlying mechanism. Accordingly, interventions targeting psychological detachment alone are unlikely to produce substantial reductions in presenteeism; a more comprehensive approach addressing additional organizational and individual-level factors is warranted.

It is worth acknowledging that the present study employed a single-mediator model, which some may consider relatively parsimonious given the complexity of the psychological mechanisms underlying the job stress–presenteeism relationship. Recent empirical work has indeed demonstrated that more intricate mediation and moderation structures may operate in occupational contexts. For instance, Li et al. (51) found that negative emotions mediated, and psychological capital moderated, the relationship between risk perception and professional commitment among nursing students during the COVID-19 pandemic; similarly, Ma et al. (52) demonstrated that career adaptability mediated the effect of perceived organisational support on career exploration among university students. These studies collectively highlight the value of examining complex boundary conditions in stress-related research. Nevertheless, the selection of psychological detachment as the sole mediator in the present study was not arbitrary, but was theoretically grounded in the Effort-Recovery Model (31) and Conservation of Resources Theory (30). Both frameworks converge on the premise that psychological detachment—defined as the cognitive and emotional disengagement from work during non-working hours—constitutes the most proximal mechanism through which job stress is transmitted to presenteeism. Specifically, sustained exposure to the hindrance-type stressors captured by the Chinese Nurses Stressor Scale (CNSS), including workload overload, role conflict, and insufficient organisational support, chronically depletes nurses’ psychological resources and impairs their capacity for recovery-oriented detachment, ultimately manifesting in presenteeism behaviours. While variables such as psychological capital and perceived organisational support may theoretically exert influence within this pathway, they function as more distal antecedent resources that shape the conditions under which detachment occurs, rather than directly transmitting the effect of job stress onto presenteeism. Furthermore, incorporating emotional exhaustion as an additional mediator would risk introducing conceptual redundancy given its substantial overlap with the resource-depletion processes already operationalized by the CNSS. The inclusion of additional moderators would require *a priori* theoretical justification and adequate statistical power beyond the scope of the present cross-sectional design. Future studies are therefore encouraged to construct moderated mediation models incorporating psychological capital or perceived organizational support as boundary conditions, so as to more comprehensively elucidate the psychological pathways through which job stress impacts presenteeism among clinical nurses.

## 5 Limitations

First, this study employs a cross-sectional design, which limits its ability to establish causal relationships and temporal precedence among variables. Although the PROCESS macro was employed to test the mediating role of psychological detachment, the assumed causal ordering—whereby job stress precedes psychological detachment, which in turn precedes presenteeism—cannot be empirically verified. Furthermore, the possibility of reverse causality cannot be ruled out—for instance, nurses with persistently high levels of presenteeism may develop heightened job stress perceptions over time due to cumulative resource depletion. Future studies should therefore adopt longitudinal or multi-wave panel designs to establish temporal sequences and validate the conclusions of this research. Second, all measurement results are based on subjective reports, which may introduce potential reporting bias and recall bias. It is recommended to incorporate objective assessments using economic indicators related to health-related productivity loss. Furthermore, the evaluation of presenteeism in this study relies on respondents’ recollection of the number of times they should have taken sick leave but chose to continue working over the past year. Due to the long recall period, respondents are susceptible to recall bias. The sample for this study was drawn from five hospitals in Sichuan Province, which may not represent the broader population of nurses in China. To enhance the generalizability and reproducibility of the research findings, future studies should consider including multi-center and cross-regional samples. Third, this study focused primarily on individual-level psychological mechanisms and did not sufficiently account for broader contextual factors—such as time allocation, work-family balance, and institutional support—that may constrain nurses’ capacity for psychological detachment (53). Future studies are encouraged to incorporate variables such as scheduling flexibility, family caregiving burden, and work-family boundary management into the theoretical framework, so as to more comprehensively elucidate the boundary conditions shaping the effectiveness of psychological detachment among clinical nurses. Furthermore, although the indirect effect of psychological detachment in the job stress–presenteeism pathway was statistically significant, the effect size was modest (*β* = 0.06), accounting for 30% of the total effect. This suggests that psychological detachment explains only a partial segment of the mechanism through which job stress influences presenteeism, and that other mediating variables—such as emotional exhaustion, sleep quality, or social support—may account for additional variance in this relationship. Future studies are encouraged to incorporate multiple mediators within an integrated theoretical framework to more comprehensively elucidate the pathways linking job stress to presenteeism among clinical nurses.

## Data Availability

Requests to access the datasets should be directed to XZ, 993294749@qq.com.
